# Large Hydatid Cyst of the Cervical Soft Tissue Extending to the Mediastinum: A Report of a Rare Case and Literature Review

**DOI:** 10.7759/cureus.106345

**Published:** 2026-04-02

**Authors:** Mirwais Safi, Hamidullah Hamid, Ehsanullah Safi, Nasrullah Shefayee, Muhibullah Saifi

**Affiliations:** 1 General Surgery, Ibn Sina Emergency Hospital, Kabul, AFG

**Keywords:** cervical hydatid disease, cyst hydatid, echinococcosis, mediastinal extension, surgical case reports

## Abstract

Hydatid disease (*Echinococcus* infection) is a parasitic condition primarily affecting the liver and lungs. Cervical involvement is extremely rare and can mimic other benign cystic neck lesions, making diagnosis challenging. We report a 35-year-old woman with a gradually enlarging left cervical mass extending toward the superior mediastinum, causing dysphagia and mild shortness of breath. Imaging revealed a large cystic lesion with daughter cysts abutting cervical vessels and the subclavian artery, with no abdominal or pulmonary involvement. The patient underwent transcervical excision with careful preservation of surrounding structures, followed by postoperative albendazole therapy. She recovered uneventfully and remains recurrence-free.

This case highlights that hydatid disease should be considered in the differential diagnosis of cystic neck masses, particularly in rural or endemic regions. Accurate preoperative imaging is essential to delineate lesion extent, especially with mediastinal extension, and meticulous surgical excision without rupture is critical to prevent complications and recurrence.

## Introduction

Hydatid disease is a zoonotic parasitic infection caused by the larval stage of *Echinococcus granulosus* [1]. It most commonly affects the liver and lungs, while involvement of other sites is relatively uncommon [1,2]. Primary cervical hydatid cysts are exceedingly rare, accounting for less than 1% of reported cases even in endemic regions [3,4]. Due to their rarity and non-specific presentation, they may mimic more common cystic neck lesions, leading to diagnostic challenges and potential delays in management [1].

This case is distinguished by its unusual cervical localization with extension into the superior mediastinum and associated compressive features, posing both diagnostic and surgical challenges. In addition to presenting this rare manifestation, we provide a focused review of the literature to contextualize its clinical features and management, thereby contributing to improved recognition and treatment of similar cases in endemic settings.

## Case presentation

A 35-year-old female farmer, involved in crop cultivation and livestock rearing, presented with a gradually enlarging left cervical mass for four years, associated with dysphagia and mild shortness of breath. She had no prior medical history or travel outside her rural community.

**Figure 1:**
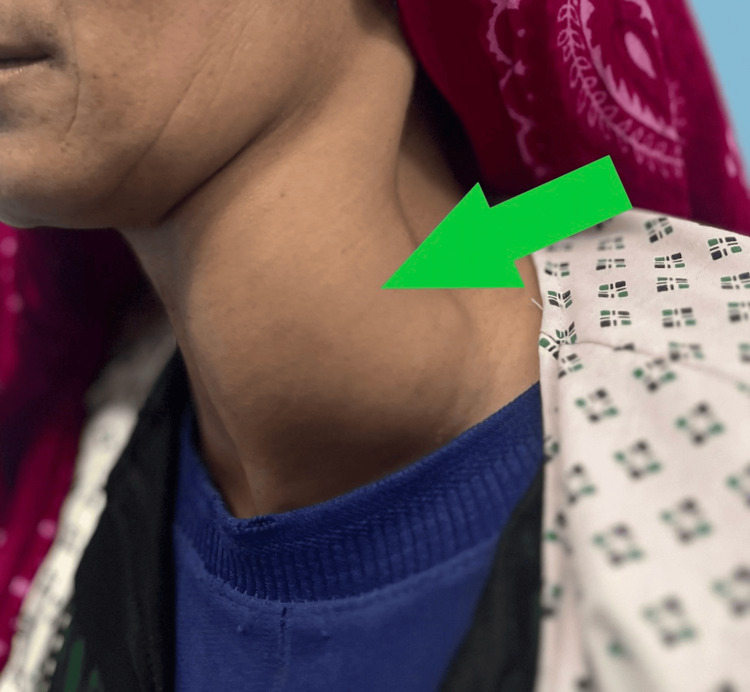
Clinical photograph showing a prominent swelling on the left side of the neck (arrow) The patient’s eyes and eyebrows are obscured to maintain anonymity.

On examination, the patient was well-nourished and in no acute distress. A soft, mobile, non-tender mass measuring ~9 × 7 cm was noted in the posterior cervical triangle, extending superiorly toward the submandibular region and inferiorly toward the superior mediastinum. There were no overlying skin changes, lymphadenopathy, or neurological deficits (Figure 1). Routine laboratory tests were within normal ranges, vital signs were normal, and serology for hydatid disease was negative.

**Figure 2:**
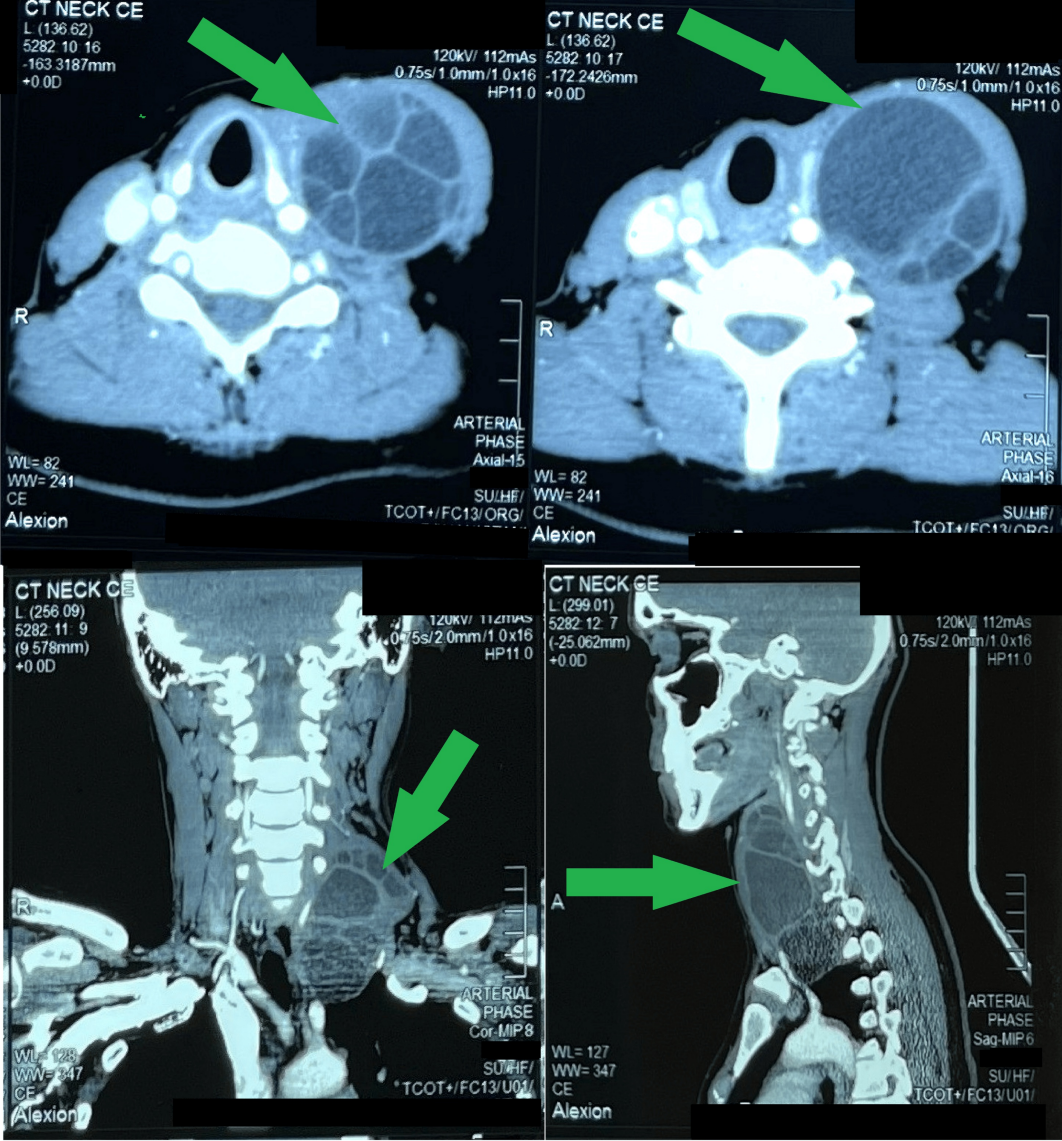
Contrast-enhanced CT images of the neck showing a well-defined multiloculated cystic lesion in the left cervical region with internal septations and daughter cysts (arrows), extending laterally and into the superior mediastinum

Ultrasonography revealed a multiloculated cystic lesion with internal septations. CT showed a 10.8 × 5 × 4.8 cm lobulated cyst posterior to the sternocleidomastoid, containing daughter cysts and abutting cervical vessels and the subclavian artery, with extension into the superior mediastinum (Figure 2). No hepatic, pulmonary, or intracranial involvement was detected. Differential diagnosis included hydatid cyst and cystic lymphangioma.

**Figure 3:**
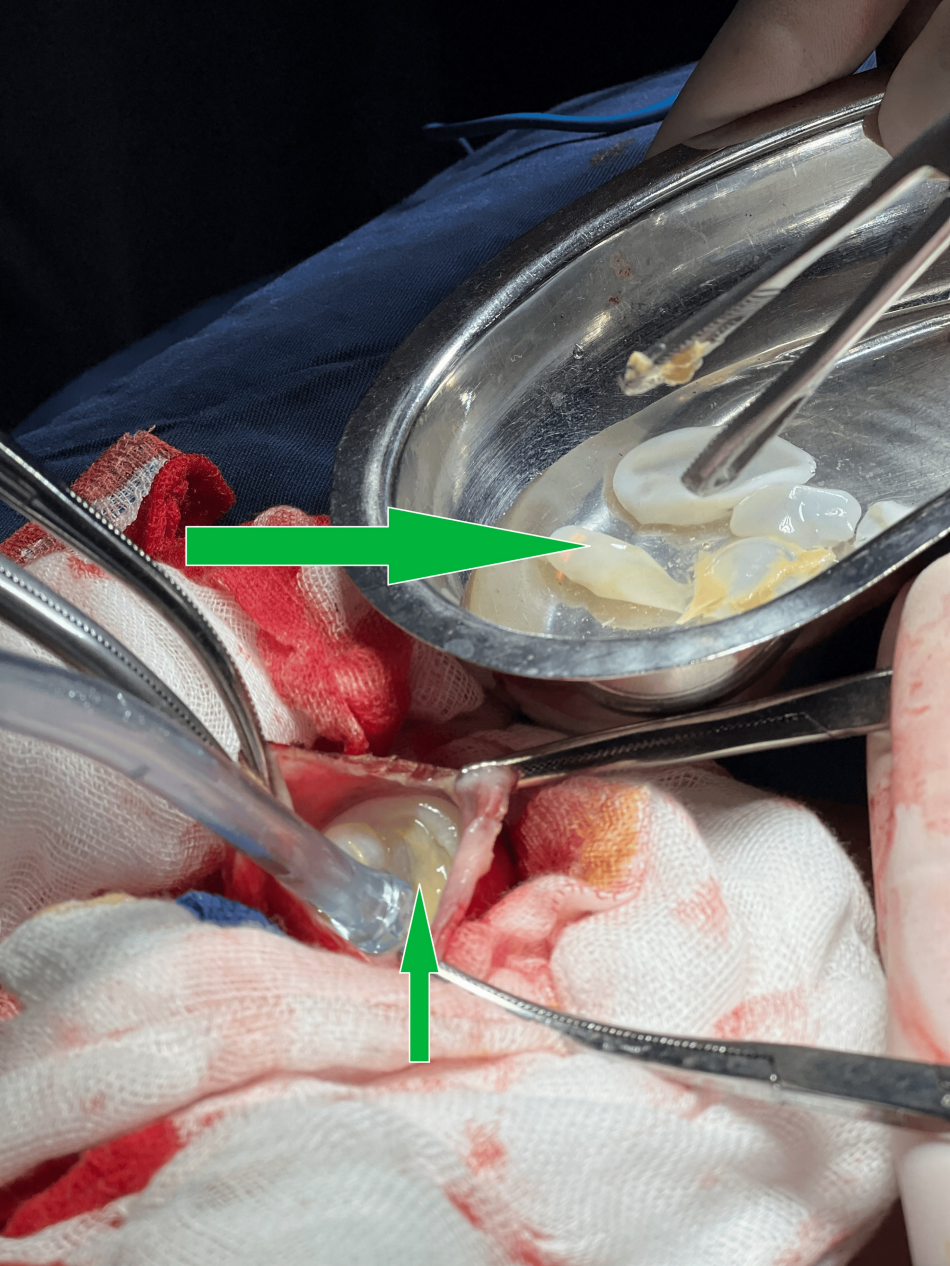
Intraoperative images of the hydatid cyst after controlled opening and evacuation The laminated membrane and daughter cysts are visible (arrows), strongly suggesting the diagnosis of hydatid disease.

The patient underwent transcervical excision. Intraoperatively, the cyst was closely adherent to cervical vessels and the left thyroid lobe. After controlled decompression, the laminated membrane and endocyst were removed, and only the superficial pericyst was excised due to dense adhesion to major vessels (Figure 3). The residual cavity was managed with capitonnage, with no intraoperative complications.

Postoperatively, she received albendazole 400 mg twice daily in a cyclic regimen (28 days on, 14 days off, for three cycles). Follow-up over seven months showed no recurrence; imaging of the abdomen and chest remained normal. Consequently, histopathology confirmed the diagnosis of a primary cervical hydatid cyst.

## Discussion

Hydatid disease, caused by *Echinococcus granulosus*, is endemic in regions such as the Middle East, India, Africa, and South Asia [1,5]. Humans are accidental intermediate hosts, typically infected via ingestion of parasite eggs through contaminated food, water, or contact with infected animals [6]. The liver and lungs are most commonly affected, although cysts can rarely occur in atypical sites, including the brain, bones, and other extra-abdominal locations [6,7]. Cervical hydatid cysts are exceptionally rare (<1% of cases) and often overlooked in the differential diagnosis of cystic neck masses [8].

Hydatid cysts usually grow slowly (~1 cm/year), and clinical manifestations depend on size, location, and complications such as infection or rupture [5,9]. Most cervical cases present as small, painless masses in superficial compartments [9]. In contrast, this patient had a large cyst with superior mediastinal extension and firm adhesion to major vessels, producing compressive symptoms (dysphagia and dyspnea), emphasizing its clinical significance and surgical complexity.

The differential diagnosis of cystic neck lesions is broad, including congenital cysts, inflammatory masses, and neoplasms such as lymphangiomas or cystic metastases [3,9,10]. Epidemiological clues, such as rural residence and close contact with livestock or dogs, support consideration of hydatid disease, particularly in endemic areas [11,12].

Imaging is central for diagnosis and surgical planning. Ultrasonography is usually first-line, while CT defines lesion extent and relation to adjacent structures, often showing daughter cysts or the “water lily sign” [3]. MRI further clarifies soft tissue involvement [13]. Systemic imaging is recommended, as multi-organ involvement occurs in 20%-40% of patients [14]. In this case, CT revealed a multiloculated cervical cyst with daughter cysts extending into the superior mediastinum and abutting major vessels, a rare finding crucial for operative planning, while abdominal and chest imaging excluded other involvement.

Serology can support diagnosis in 80%-90% of cases but may be negative in extrahepatic lesions [13]. Fine-needle aspiration is generally avoided due to risk of rupture, dissemination, and anaphylaxis (~1% complication rate) [15]; this patient’s serology was negative.

Cervical hydatid cysts have been reported in the parotid, lateral cervical, thyroid, submental, and submandibular regions [16,17]. Most remain confined to the neck, whereas mediastinal extension is exceedingly rare and poses surgical challenges [11]. Excision with adjunctive albendazole remains the standard, aiming for complete removal without rupture [4]. Dense vascular adhesions may necessitate partial pericystectomy [7], and some cases require combined cervicothoracic approaches [18]. In this patient, transcervical excision alone was feasible despite mediastinal extension, highlighting that less invasive approaches can be successful with careful planning.

The role of intraoperative scolicidal agents remains debated, particularly in sensitive regions where chemical injury risk must be weighed against recurrence prevention [19]. Albendazole regimens vary from short perioperative courses to prolonged cyclic therapy [20]; here, a 28-day on/14-day off cycle for three cycles was used, with no recurrence, consistent with commonly adopted protocols [3].

This case is distinguished by large cyst size, superior mediastinal extension, and vascular proximity, requiring partial pericystectomy. It underscores the importance of individualized surgical planning and adjunctive therapy in complex cervical hydatid cysts.

A summary of previously reported cases is provided in Table 1, highlighting the rarity of mediastinal extension, compressive symptoms, and the spectrum of clinical presentations.

**Table 1:** Summary of reported cases of cervical hydatid cysts, comparatively highlighting key clinical features; the present case is distinguished by mediastinal extension, compressive symptoms, and the need for partial pericystectomy

Author(Reference)	Age/Sex	Location	Size in cm	Mediastinal extension	Compressive Symptoms	Treatment	Outcome	Pericystectomy (Total/Partial)
[1]	20/male	right upper neck area	3.3 × 4.5	NO	NO	Surgical excision	Uneventful	Total
[3]	29/male	left posterior triangle	5 × 4	NO	left upper limb paresthesia	Surgical excision + albendazole	Uneventful	Total
[4]	6/female	left postauricular	3.5 × 3	No	No	Surgical excision + Albendazole	Uneventful	Total
[5]	6/male	left side of the neck	6	NO	Trouble breathing	Surgical excision + Albendazole	Uneventful	Total
[7]	40/male	right supraclavicular fossa	5	NO	No	Surgical excision	Uneventful	Total
[9]	3/male	left side of the neck	3.5	No	NO	Surgical excision	Uneventful	Total
[11]	26/female	Ant. Neck	7.5 x 5 x 5	yes	Dysphagia and shortness of breath	Surgical excision	Uneventful	Total + total thyroidectomy
[13]	20/female	posterior cervical	7x 7x4	NO	No	Surgical excision + Albendazole	Uneventful	Total
[16]	54/male	left parotid gland	3	No	NO	Surgical excision	Uneventful	Total + Lt. parotidectomy
[17]	13/female	right submandib-ular	5 × 4.5	No	No	Surgical excision + Albendazole	Uneventful	Total
This case	35/female	left posterior neck	10.8 × 5 × 4.8	Yes	dysphagia and mild shortness of breath	Surgical excision + Albendazole	Uneventful	Partial pericystectomy

## Conclusions

Primary cervical hydatid cysts are rare, and this case is notable for mediastinal extension and proximity to major neurovascular structures, which increased surgical complexity. Clinicians should consider hydatid disease in the differential diagnosis of cystic neck masses, especially in endemic areas. Careful preoperative imaging and meticulous surgical excision, complemented by postoperative anthelminthic therapy, are crucial to prevent complications and recurrence. This case emphasizes individualized surgical planning for anatomically challenging presentations.
